# Co-infection and localization of secondary symbionts in two whitefly species

**DOI:** 10.1186/1471-2180-10-142

**Published:** 2010-05-12

**Authors:** Marisa Skaljac, Katja Zanic, Smiljana Goreta Ban, Svetlana Kontsedalov, Murad Ghanim

**Affiliations:** 1 Department of Applied Sciences (Plant Protection), Institute for Adriatic Crops, Split, Croatia; 2 Department of Entomology, Institute Plant Protection, Agricultural Research Organization, The Volcani Center, Bet Dagan, Israel

## Abstract

**Background:**

Whiteflies are cosmopolitan phloem-feeding pests that cause serious damage to many crops worldwide due to direct feeding and vectoring of many plant viruses. The sweetpotato whitefly *Bemisia tabaci *(Gennadius) and the greenhouse whitefly *Trialeurodes vaporariorum *(Westwood) are two of the most widespread and damaging whitefly species. To complete their unbalanced diet, whiteflies harbor the obligatory bacterium *Portiera aleyrodidarum. B. tabaci *further harbors a diverse array of secondary symbionts, including *Hamiltonella, Arsenophonus, Cardinium, Wolbachia, Rickettsia *and *Fritschea*. *T. vaporariorum *is only known to harbor *P. aleyrodidarum *and *Arsenophonus*. We conducted a study to survey the distribution of whitefly species in Croatia, their infection status by secondary symbionts, and the spatial distribution of these symbionts in the developmental stages of the two whitefly species.

**Results:**

*T. vaporariorum *was found to be the predominant whitefly species across Croatia, while only the Q biotype of *B. tabaci *was found across the coastal part of the country. *Arsenophonus *and *Hamiltonella *were detected in collected *T. vaporariorum *populations, however, not all populations harbored both symbionts, and both symbionts showed 100% infection rate in some of the populations. Only the Q biotype of *B. tabaci *was found in the populations tested and they harbored *Hamiltonella*, *Rickettsia, Wolbachia *and *Cardinium*, while *Arsenophonus *and *Fritschea *were not detected in any *B. tabaci *populations. None of the detected symbionts appeared in all populations tested, and multiple infections were detected in some of the populations. All endosymbionts tested were localized inside the bacteriocyte in both species, but only *Rickettsia *and *Cardinium *in *B. tabaci *showed additional localization outside the bacteriocyte.

**Conclusions:**

Our study revealed unique co-infection patterns by secondary symbionts in *B. tabaci *and *T. vaporariorum*. Co-sharing of the bacteriocyte by the primary and different secondary symbionts is maintained through vertical transmission via the egg, and is unique to whiteflies. This system provides opportunities to study interactions among symbionts that co-inhabit the same cell in the same host: these can be cooperative or antagonistic, may affect the symbiotic contents over time, and may also affect the host by competing with the primary symbiont for space and resources.

## Background

**Figure 1:**
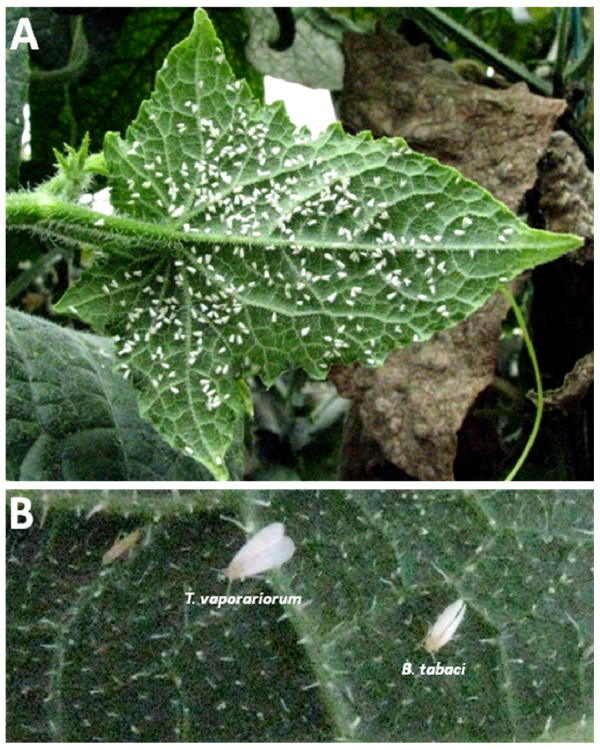
**Whiteflies in Croatia**. Demonstration of heavy whitefly infestations on cucumbers grown in the coastal part of Croatia (A), and external phenotypical differences between *B. tabaci *and *T. vaporariorum *(B).

Whiteflies (Hemiptera: Aleyrodidae) are an extremely important group of agricultural insect pests that cause serious damage by weakening plants, excreting honeydew and transmitting several hundreds of plant viruses [1]. The most economically important of these is the cosmopolitan sweetpotato whitefly *Bemisia tabaci *(Gennadius), which is a species complex of more than 20 biotypes. The B and Q biotypes, among the most predominant and damaging worldwide, differ in many biological parameters, including resistance to insecticides, ability to damage plants [2] and tolerance to environmental conditions [3]. Another important whitefly insect pest is the greenhouse whitefly *Trialeurodes vaporariorum *(Westwood) which is less important as a virus vector, but causes serious damage by direct feeding on plants. Whereas *T. vaporariorum *can be identified based on external morphology (Figure 1), *B. tabaci *biotypes are only well defined by DNA markers [4].

Symbiosis is quite common among known whitefly species. Both *B. tabaci *and *T. vaporariorum *harbor the primary obligatory bacterium *Portiera aleyrodidarum*, which supplements their unbalanced diet [5]. *B. tabaci *can also harbor a diverse array of facultative secondary symbionts, including the Gammaproteobacteria *Arsenophonus *(Enterobacteriales), *Hamiltonella *(Enterobacteriales) [5,6], *Fritschea *(Chlamydiales) [7] and *Cardinium *(Bacteroidetes) [8], and the Alphaproteobacteria *Rickettsia *(Rickettsiales) [9] and *Wolbachia *(Rickettsiales) [10]. A clear association between *B. tabaci *biotypes and secondary symbionts has been observed in Israeli populations: *Hamiltonella *is detected only in the B biotype, *Wolbachia *and *Arsenophonus *only in the Q biotype, and *Rickettsia *in both biotypes [11]. *Fritschea *has only been detected in the A biotype from the United States [12], and only *Arsenophonus *has been associated with *T. vaporariorum *[13]. Virtually nothing is known about the functions these symbionts might fulfill in whiteflies. However, in other arthropods, they may influence their host's nutrition, host plant utilization and ability to cope with environmental stress factors, induce resistance to parasitoids, and effect reproductive manipulations [14]. For example *Wolbachia, Cardinium*, *Rickettsia *and *Arsenophonus *are known to manipulate reproduction in a wide range of insect species by inducing cytoplasmic incompatibilities or sex ratio bias [15-18]. *Hamiltonella defensa *induces parasitoid resistance in the pea aphid [19], whereas *Fritschea bemisiae *has no known effect. Recent studies have shown that different single and mixed infections with secondary symbionts in *B. tabaci *can affect the insects' ability to tolerate synthetic pesticides [20,21]. The diversity and infection status of other world whitefly populations have not been documented. In the framework of a large study to identify the status of whitefly pests in Croatia, we describe the distribution of whitefly populations in that country, their infection status by secondary symbionts, co-infections and spatial localization within the insects' developmental stages. Interestingly, infection with secondary symbionts and localization patterns in *B. tabaci *differed in some cases from previously published results. In *T. vaporariorum*, this is the first time in which such a study has been reported.

## Results

### *B. tabaci *distribution and infection by secondary symbionts

**Figure 2:**
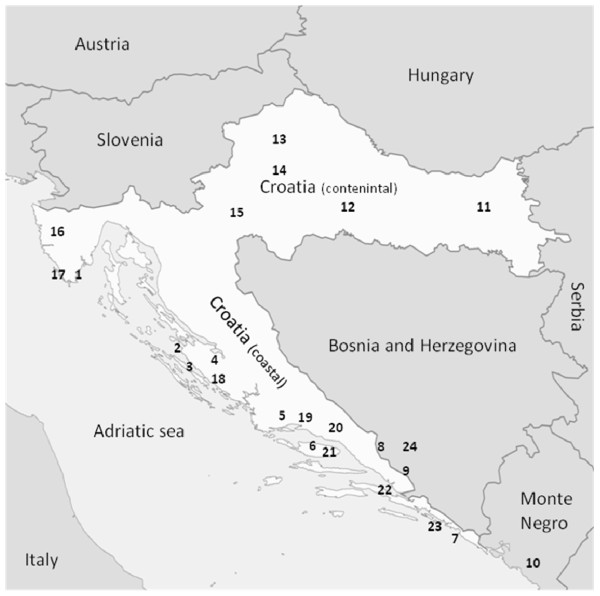
**Distribution of whiteflies in Croatia**. Locations of the populations collected in this study in Croatia and neighboring countries. Names of locations are given in Table 1.

**Figure 3:**
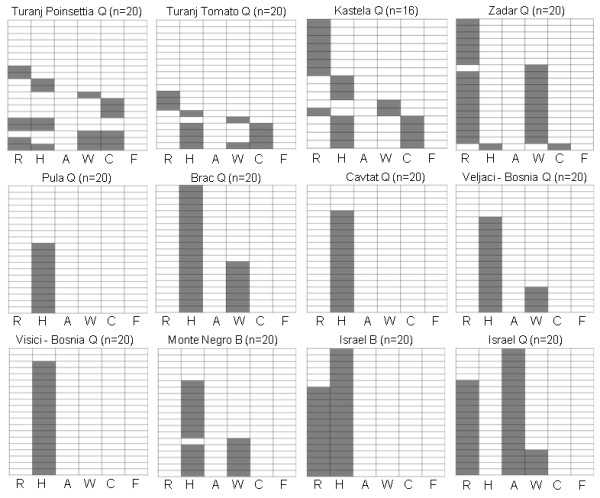
**Individual and mixed infections by secondary symbionts in *B. tabaci *populations collected in this study**. 10 populations from Croatia were tested, and two additional populations from Israel were tested for comparison. Each box represents one population. Vertical columns represent the different symbionts tested as indicated in the base of each column, and each horizontal column represents one individual that was tested for the presence of the six different symbionts. Gray shading represent positive infection with the tested symbiont. The geographical origin of the population, the biotype and the number of individuals tested are indicated at the top of each box. (R) *Rickettsia*, (H) *Hamiltonella*, (A) *Arsenophonus*, (W) *Wolbachia*, (C) *Cardinium*, (F) *Fritschea*.

Whitefly collections in Croatia were conducted in 2008-2009. Ten *B. tabaci *populations (Table 1) were collected only from the coastal part of the country because, surprisingly, *B. tabaci *was never found inland (the continental part), presumably due to the different climates (Figure 2). Interestingly, testing the collected populations revealed only the Q biotype. One population collected in the neighboring Monte Negro was identified as B biotype. Twenty individuals from each population were tested for the presence of the different symbionts known from whiteflies using genus-specific primers for each symbiont (Table 2). *P. aleyrodidarum*, the primary symbiont, was detected in all individuals tested and provided a control for the quality of the extracted DNA. Each box in Figure 3 shows single and mixed infections detected in all of the individuals in a population. For example, the population collected from Turanj on poinsettia plants (population 4 in Table 1) contained only two individuals that were singly infected with *Rickettsia*, two individuals that harbored only *Hamiltonella*, one individual that harbored only *Wolbachia *and three individuals that harbored only *Cardinium*. This population also contained two individuals that were doubly infected with *Rickettsia *and *Hamiltonella*, one individual that was doubly infected with *Wolbachia *and *Cardinium*, one individual that was infected with three symbionts--*Rickettsia, Wolbachia *and *Cardinium*, and one individual that showed the highest level of mixed infection with four symbionts--*Rickettsia, Hamiltonella, Wolbachia *and *Cardinium*. Among the 20 individuals tested from this location, seven did not contain any of the tested secondary symbionts. *Fritschea *was not detected in this or any other population tested in this study. Although the population from Turanj showed a high level of mixed infection, with one individual harboring four different symbionts, mixed infections with more than one symbiont were not common in many of the tested populations. All populations harbored at least one symbiont or more in some of the individuals tested, and overall, secondary symbionts were highly prevalent with 82% (194/236) of the individuals having at least one symbiont. *Hamiltonella *showed the highest prevalence in all populations tested and was detected in 52% of the individuals tested; sometimes it was the only symbiont detected in a particular population and it was fixed or close to fixation in some populations, for example those collected in Pula, Cavtat and Visici. The presence of each symbiont varied considerably between populations. For example *Hamiltonella *was fixed in the population from Brac, and this population did not harbor *Rickettsia*. However, in the population from Zadar, *Hamiltonella *was found in only one individual while *Rickettsia *was almost fixed. Single infections were more prevalent (52% of the total individuals tested) than mixed infections (two or more symbionts in the same individual--31% of all individuals tested). All symbionts tested were found in at least one or more cases in which they were co-infecting the same individual. Figure 3 demonstrates the high variability in secondary symbiont prevalence in the different populations tested, and while some populations were heterogeneous and contained multiple symbionts (for example the populations from Turanj), other populations were found to be infected with only one symbiont (the populations from Pula and Cavtat).

**Table 1:** *B. tabaci *and *T. vaporariorum *populations collected across Croatia and neighboring countries in this study

Population number	Collection location	Species and biotype	Host plant
1	Pula	*B. tabaci *Q	Poinsettia
2	Zadar	*B. tabaci *Q	Hibiscus
3	Turanj	*B. tabaci *Q	Tomato
4	Turanj	*B. tabaci *Q	Poinsettia
5	Kastela	*B. tabaci *Q	Hibiscus
6	Brac	*B. tabaci *Q	Cucumber
7	Cavtat	*B. tabaci *Q	Black nightshade
8	Veljaci (Bosnia and Herzegovina)	*B. tabaci *Q	Zucchini
9	Visici (Bosnia and Herzegovina)	*B. tabaci *Q	Datura
10	Podgorica (Monte Negro)	*B. tabaci *B	Hibiscus
11	Cepin	*T. vaporariorum*	Gerbera
12	Velika Ludina	*T. vaporariorum*	Datura
13	Zabok	*T. vaporariorum*	Pumpkin
14	Donja Lomnica	*T. vaporariorum*	Strawberries
15	Karlovac	*T. vaporariorum*	Zucchini
16	Novigrad	*T. vaporariorum*	Tomato
17	Pula	*T. vaporariorum*	Petunia
18	Turanj	*T. vaporariorum*	Tomato
19	Split	*T. vaporariorum*	Tobacco
20	Tugare	*T. vaporariorum*	Cucumber
21	Brac	*T. vaporariorum*	Cucumber
22	Metkovic	*T. vaporariorum*	Tomato
23	Dubrovnik	*T. vaporariorum*	Gerbera
24	Veljaci (Bosnia and Herzegovina)	*T. vaporariorum*	Cucumber

**Table 2:** List of primers used in this study

Targeted gene	Primer name	Sequence (5'-> 3')	Anealing (°C)/Product Size	Reference
*Rickettsia* 16S rDNA	Rb-F Rb-R	GCTCAGAACGAACGCTATC GAAGGAAAGCATCTCTGC	59/~900	[9]
				
*Hamiltonella* 16S rDNA	92F HbR	TGAGTAAAGTCTGGGAATCTGG AGTTCAAGACCGCAACCTC	62/~700	[10]
				
*Cardinium* 16S rDNA	CFB-F CFB-R	GCGGTGTAAAATGAGCGTG ACCTMTTCTTAACTCAAGCCT	59/~500	[8]
				
*Arsenophonus* 23S rDNA	Ars23S-1 Ars23S-2	CGTTTGATGAATTCATAGTCAAA GGTCCTCCAGTTAGTGTTACCCAAC	59/~600	[5]
				
*Wolbachia* 16S rDNA	Wol16S-f Wol16S-r	CGG GGGAAAAATTTATTGCT AGCTGTAATACAGAAAGTAAA	55/~650	[38]
				
*Fritschea* 16S rDNA	U23F 23SIGR	GATGCCTTGGCATTGATAGGCGATGAAGGA TGGCTCATCATGCAAAAGGCA	55/~600	[7]
				
B/Q biotypes mtCO1	C1-J-2195 L2-N-3014	TTGATTTTTTGGTCATCCAGAAGT TCCAATGCACTAATCTGCCATATTA	50/~850	[57]
				
B/Q biotypes micro-sattelite	Bem23-F Bem23-R	CGGAGCTTGCGCCTTAGTC CGGCTTTATCATAGCTCTCGT	55/Q = 400 B = 200	[56]

### *T. vaporariorum *distribution and infection by secondary symbionts

**Figure 4:**
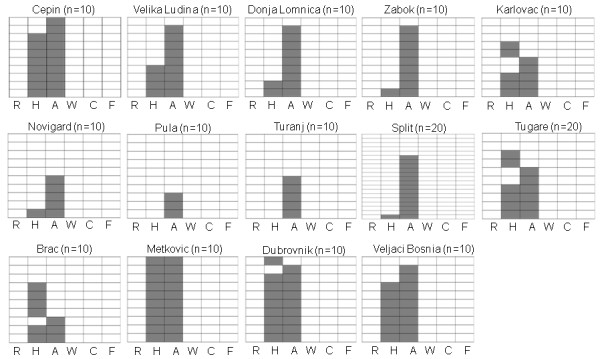
**Individual and mixed infection by secondary symbionts in *T. vaporariorum *populations collected in this study**. (14 populations were tested). See legend to Figure 3.

Fourteen *T. vaporariorum *populations were collected across Croatia's coastal and continental regions as well as from neighboring Bosnia and Herzegovina and tested for the presence of secondary symbionts. *T. vaporariorum *was much more prevalent than *B. tabaci *in most of the regions, sometimes with heavy infestations in agricultural crops. *P. aleyrodidarum*, the primary symbiont, was detected in all individuals tested. Out of the six secondary symbionts tested in the collected *T. vaporariorum *populations, only *Arsenophonus *and *Hamiltonella *were detected (Figure 4). *Arsenophonus *was more prevalent than *Hamiltonella*: it appeared in 71% of all individuals tested (107/150), as a single infection in 37% of all individuals, while the latter was detected in 40% of all individuals, and appeared as a single infection in 6% of all individuals (Figure 4). The prevalence of *Arsenophonus *was always higher or equal to that of *Hamiltonella *in all populations tested except for the population from the island Brac. Two of the populations tested were not infected with *Hamiltonella *(Pula and Turanj) and one population showed fixation of both symbionts (Metkovic); 34% (51/150) of all individuals tested were doubly infected with *Arsenophonus *and *Hamiltonella *(Figure 4).

### Localization of secondary symbionts in *B. tabaci *and *T. vaporariorum*

None of the controls used with the samples submitted to fluorescence in situ hybridization (FISH) showed any signal (data not shown). All tested symbionts varied in their localization pattern, which could be divided into two types: total confinement to the bacteriocyte during all developmental stages, and confinement combined with scattered localization outside the bacteriosome during some of the developmental stages.

**Figure 5:**
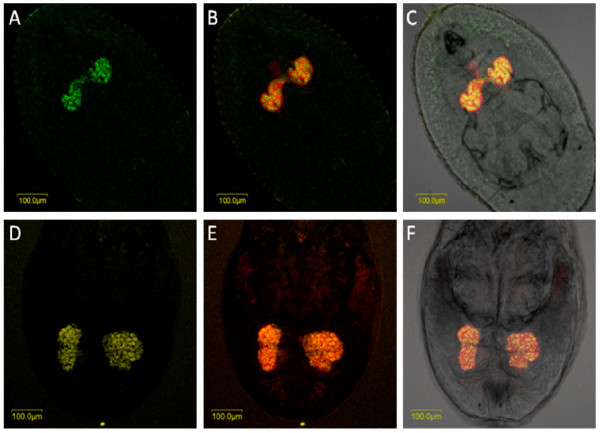
***Portiera, Arsenophonus *and *Hamiltonella *FISH of *T. vaporariorum *nymphs**. *Portiera*-specific probe (red) and probes specific to secondary symbionts *Hamiltonella *(green) and *Arsenophonus *(yellow) were used. A-C: FISH of *Hamiltonella *alone (A), double FISH of *Hamiltonella *and *Portiera *under dark field (B), and double FISH of *Hamiltonella *and *Portiera *under bright field (C). D-F: FISH of *Arsenophonus *alone (D), double FISH of *Arsenophonus *and *Portiera *under dark field (E), and double FISH of *Arsenophonus *and *Portiera *under bright field (F).

**Figure 6:**
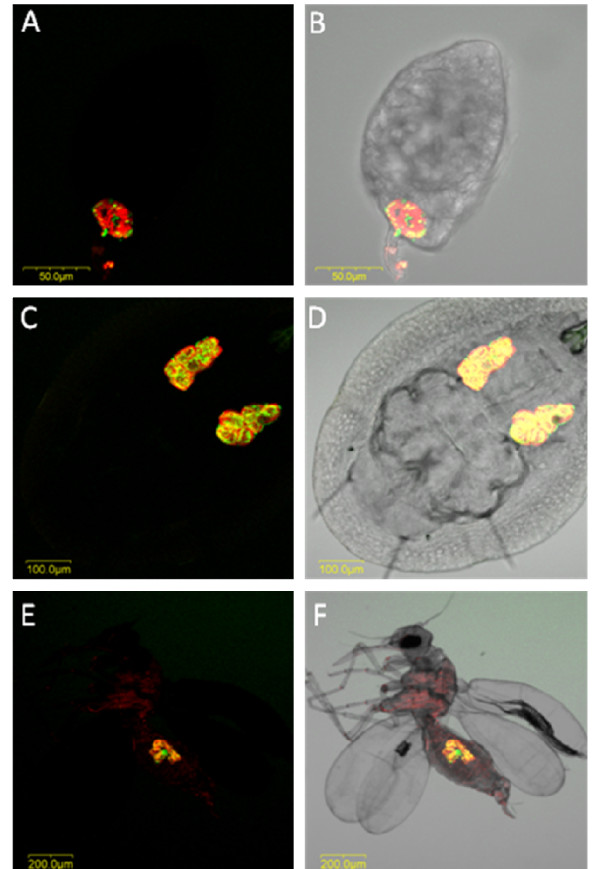
***Portiera *and *Hamiltonella *FISH of *B. tabaci *eggs, nymphs and adults**. *Portiera*-specific probe (red) and *Hamiltonella*-specific probe (green) were used. A, C and E: double FISH of *Portiera *and *Hamiltonella *in eggs (A), nymphs (C) and adults (E) under dark field. B, D and F: double FISH of *Portiera *and *Hamiltonella *in eggs (B), nymphs (D) and adults (F) under bright field.

**Figure 7:**
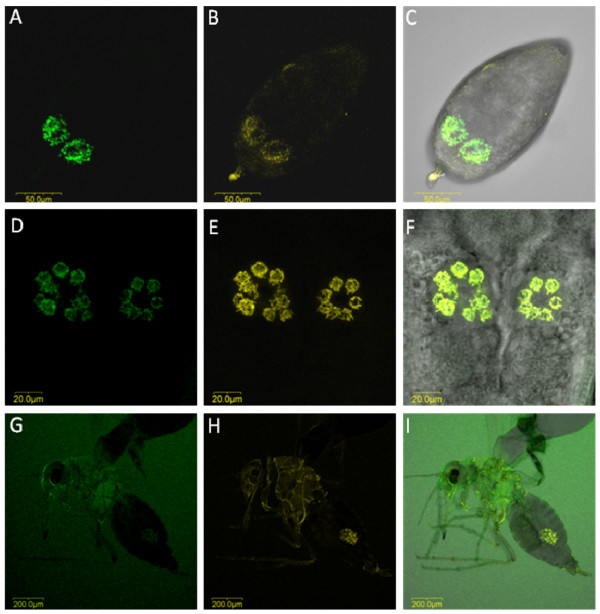
***Hamiltonella *and *Arsenophonus *FISH of *T. vaporariorum *eggs, nymphs and adults**. Secondary symbiont-specific probes for *Hamiltonella *(green) and *Arsenophonus *(yellow) were used. A, D and G: FISH of *Hamiltonella *alone in eggs (A), nymphs (D) and adults (G). B, E and H: FISH of *Arsenophonus *alone in eggs (B), nymphs (E) and adults (H). C, F and I: double FISH of *Hamiltonella *and *Arsenophonus *in eggs (C), nymphs (F) and adults (I).

*Hamiltonella *was localized to small areas inside the bacteriocyte: these areas appeared sometimes as independent and homogenous small patches as in *T. vaporariorum *(Figure 5A-C) and sometimes continuous and irregular as in *B. tabaci *(Figure 6). These patterns of localization were observed in eggs, nymphs and adults of both *T. vaporariorum *and *B. tabaci *(Figs. 5A-C and 6). The pattern of localization of *Arsenophonus *in *T. vaporariorum *was similar to that of *Hamiltonella *(Figure 5D-F). Both symbionts always co-localized with *Portiera *which occupied most of the bacteriocyte. The continuous and irregular localization phenotype of *Hamiltonella *has been previously observed in *B. tabaci *by FISH and TEM [22]; however the phenotype in *T. vaporariorum *is different. *Hamiltonella *and *Arsenophonus *were never observed outside the bacteriocyte. Sequencing of 900 bp of the 16S rRNA *Hamiltonella *gene from *T. vaporariorum *showed 95% similarity with *B. tabaci Hamiltonella *(data not shown). Interestingly, *Arsenophonus *always co-localized to exactly the same areas with *Hamiltonella*, in eggs, nymphs and adults of *T. vaporariorum *(Figure 7). Previously described *B. tabaci *Q biotype populations have never been reported to harbor *Hamiltonella*; however, those populations were infected with *Arsenophonus *at high rates, and thus the two symbionts could not be observed in the same individual. Conversely, *Arsenophonus *was not observed in any of the *B. tabaci *populations collected in this study, which did harbor *Hamiltonella*. Thus these two endosymbionts never co-localized in the same *B. tabaci *individual, whereas they co-localized in *T. vaporariorum*. The localization pattern of *Arsenophonus *in *T. vaporariorum *also resembled that of its previously published localization in *B. tabaci *[22], and it was observed to be rod-shaped, in agreement with TEM and light microscopic images of cell lines infected with this bacterium [23].

**Figure 8:**
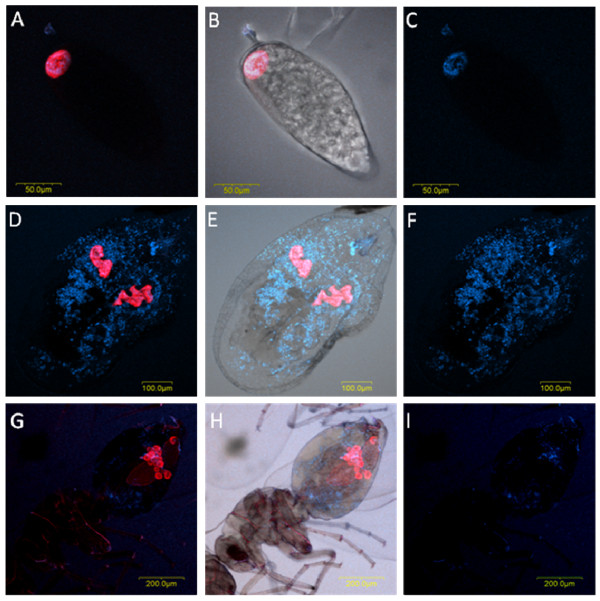
***Portiera *and *Cardinium *FISH of *B. tabaci *eggs, nymphs and adults**. *Portiera*-specific probe (red) and *Cardinium*-specific probe (blue) were used. A, C and G: double FISH of *Portiera *and *Cardinium *in eggs (A), nymphs (D) and adults (G) under dark field. B, E and H: double FISH of *Portiera *and *Cardinium *in eggs (B), nymphs (E) and adults (H) under bright field. C, F and I are shown only with *Cardinium *probe to emphasize its location inside the bacteriosome.

*Cardinium *showed a dual localization pattern, outside and inside the bacteriocyte, with *Portiera *in the same *B. tabaci *individuals (Figure 8). *Cardinium*, like all symbionts that are confined to the bacteriocyte, is transovarially transferred from the mother to the offspring though the egg. Thus in the egg's early developmental stages, it is confined to the bacteriocyte; however, in older eggs (5-7 days), it is also observed outside the bacteriocyte (not shown), and in later nymphal and adult stages, it occupies most of the body tissues, including the bacteriocyte (Figure 8). *Cardinium *was not detected in *T. vaporariorum. Cardinium *has been shown by TEM to localize to the bacteriocytes of the A and Jatropha biotypes of *B. tabaci *[24]. Our PCR screening assay revealed co-localization of *Cardinium *in *B. tabaci *populations (in 15 out of a total 236 individuals tested), mostly with *Hamiltonella *(10 of the 15 *Cardinium*-containing individuals also harbored *Hamiltonella*--66% co-localization). In some cases, multiple infections of *Cardinium *with two (*Wolbachia *and *Rickettsia*) or three (*Rickettsia, Wolbachia *and *Hamiltonella*) symbionts were observed. The localization pattern of *Cardinium *as seen by FISH was different from that of the other symbionts that co-localized with it. Localization of *Hamiltonella *and *Cardinium *has also been demonstrated in the bacteriocytes of the A biotype together with *Portiera*, as shown here. TEM has revealed the presence of *Cardinium *in the spermatid cytoplasm, residual bodies, and cyst cell cytoplasm of *B. tabaci *males [25]. Studies on other hosts have reported the presence of *Cardinium *in a diverse array of tissues, including the reproductive tract [26], fat bodies, and salivary glands [27,28], as well as inside bacteriocytes surrounded by oogonia in the apical region of the ovary [29].

**Figure 9:**
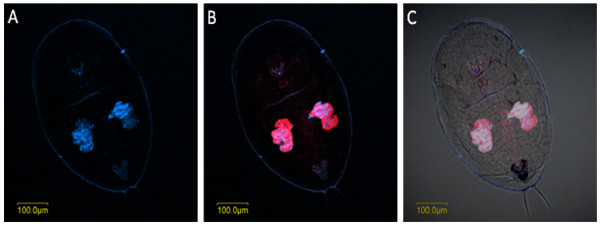
***Portiera *and *Wolbachia *FISH of *B. tabaci *nymphs**. *Portiera*-specific probe (red) and *Wolbachia*-specific probe (blue) were used. A: single FISH of *Wolbachia *under dark field, B: double FISH of *Wolbachia *and *Portiera *under dark field, C: double FISH of *Wolbachia *and *Portiera *under bright field.

*Wolbachia *has been previously shown to localize at the circumference of and inside the bacteriocytes. In adults, *Wolbachia *can also be seen in the abdomen outside the bacteriocyte [22]. Surprisingly, in our FISH analysis, *Wolbachia *could only be detected inside the bacteriocytes with the primary symbiont, and signal was not detected in any other organ at any developmental stage (Figure 9 shows the results from nymphs). The localization signal was evenly distributed in the bacteriocyte cells, but it was stronger at the cell's circumference. This different localization pattern suggests the presence of a different strain of *Wolbachia *in Croatian *B. tabaci *populations. In other insects, *Wolbachia *has been localized to organs other than the bacteriocytes, including the salivary glands, gut, Malpighian tubules, fat body and brain [30-32]. *Wolbachia *has been shown to influence the reproduction of its host and to localize to ovarian cells and developing embryos [33-35]. The localization pattern here suggests different functions for *Wolbachia *in *B. tabaci*. In our PCR screens, *Wolbachia *co-localized with one or more of the symbionts--with *Cardinium *alone, with *Cardinium *and *Rickettsia *in some individuals, with *Cardinium *and *Hamiltonella *or with *Hamiltonella, Cardinium *and *Rickettsia*. It could also be detected as a single infection. In other insects, *Wolbachia *has been found localized with other bacteria: in the aphid *Cinara cedri*, it has been found in the bacteriocytes together with *Serratia symbiotica*, and in the weevil *Sitophilus oryzae*, it co-exists with the primary symbiont [36,37].

**Figure 10:**
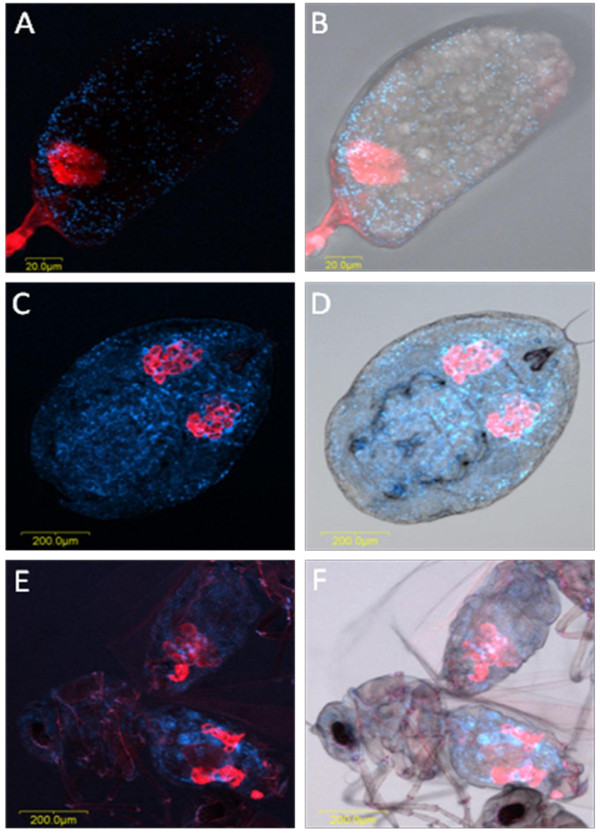
***Portiera *and *Rickettsia *FISH of *B. tabaci *eggs, nymphs and adults**. *Portiera*-specific probe (red) and *Rickettsia*-wspecific probe (blue) were used. A, C and E: double FISH of *Portiera *and *Rickettsia *in eggs (A), nymphs (C) and adults (E) under dark field. B, D and F: double FISH of *Portiera *and *Rickettsia *in eggs (B), nymphs (D) and adults (F) under bright field.

*Rickettisa *is vertically transferred with the primary symbiont into the newly developing egg. Once the new bacteriocyte cell enters the mature developing egg, it moves towards the center of the egg, and *Rickettsia *leaves it and occupies most of the egg cavity (Figure 10) [9,38]. At later stages (nymphs and adults), it is found throughout the body, except in the bacteriocytes. In the confined phenotype, *Rickettsia *is always associated with the bacteriocyte and never observed outside it. In this study, we never observed the confined phenotype, and *Rickettsia *distribution in the eggs was similar to previously published results [9]. However, in the nymphal stage, *Rickettsia *appeared to be localized inside and outside the bacteriocytes (Figure 10C). In this phenotype, *Rickettsia *cells were mostly concentrated at the circumference of the bacteriocyte cells with some sort of adhesion. Furthermore, in adults, a much higher concentration of *Rickettsia*-associated signal was consistently observed near and around the bacteriocytes relative to the rest of the body. *Rickettsia *could also be observed in the head, thorax and abdomen.

## Discussion

This study presents a comprehensive survey of the two most widespread whitefly species in Croatia, *T. vaporariorum *and *B. tabaci*, and their infection status by secondary symbionts. Their geographical distribution (Figure 2) was such that *B. tabaci *was not found in the continental part of the country. This is most likely due to climate differences between the coastal and continental parts. *T. vaporariorum*, however, was collected from all parts of the country. *B. tabaci *was found to harbor *Rickettsia, Wolbachia, Cardinium *and *Hamiltonella*, whereas *T. vaporariorum *harbored only *Arsenophonus *and *Hamiltonella*. Thus *Hamiltonella *was the only endosymbiont common to both whitefly species. Sequences of the 16S rRNA gene of *Hamiltonella *from the different *B. tabaci *populations tested in this study were identical as was the case with sequences of the same gene from all *T. vaporariorum *populations. Comparing the sequences of the 16S rRNA gene from *Hamiltonella *of both whitefly species revealed 95% similarity. This high similarity suggests different strains of *Hamiltonella *that colonize both whitefly species, however, ancient occurrence of horizontal transfer between the two species, after which *Hamiltonella *became localized to the bacteriocyte, cannot be excluded. These two whitefly species feed through the plant phloem and share host plants (Figure 1), and horizontal transmission can therefore occur through the host [33,39]. Furthermore, whiteflies share host plants with other phloem-feeders such as aphids, planthoppers and leafhoppers, which are also known to harbor endosymbionts [33,39,40]. These insects can inject endosymbionts into the vascular system which then follow the circulative pathway of transmission, reaching the salivary glands of the insect which might be involved in transmitting these symbionts [41]. A recent study has shown that salivary glands can indeed be infected by endosymbionts, as in the case of *Cardinium *in *Scaphoideus titanus *[26,42].

It is difficult to hypothesize how infections with symbionts occurred among whiteflies on an evolutionary scale: it might have been the result of horizontal transmission, loss or new acquisition of symbionts, which would partially explain the mixed infections and heterogeneity among some of the collected populations. Some populations showed very low infection rates or lacked some of the symbionts, suggesting the recent introduction of those symbionts into the populations, possibly through horizontal transfer or introduction of new whitefly populations with new symbiotic complements into Croatia via regular trade of plants. For example, among the 20 individuals tested in the Zadar population, only one individual showed infection with *Hamiltonella *and *Cardinium*. The multiple infections observed among some of the populations, such as those from Turanj and Kastela, can also be explained by efficient horizontal transfers, which allowed the appearance of maximum symbionts in one population. However, some other internal factors may influence maximum horizontal transfers and maximum infection rates in the same individuals. These factors include competition for space and resources among two or more symbionts [22,43], or on the contrary, positive interaction between the symbionts may contribute to maximum infection in one individual [44]. Another important factor is the host response to the presence of these symbionts which in most cases will influence the bacterial community residing within the host.

The occurrence of mixed infections in both species also suggests that these secondary symbionts are non-essential for these whiteflies, allowing their presence to be variable. In one report, *Hamiltonella *was found in 40% of *B. tabaci *populations [45], and 0 to 40% of pea aphid populations have been found to harbor *Rickettsia *[45-50]. Only *Hamiltonella *was highly prevalent in *B. tabaci *populations and sometimes reached fixation, an indication of a mutualistic or obligatory interaction with the insect. Such interactions can occur via complementation of the primary symbiont's function with regard to completing the host's dietary needs or enhancing host fitness.

All of the symbionts detected in both whitefly species were located together with the primary symbiont *Portiera *in the bacteriocytes at one or more stages of development. However, some were strictly localized to the bacteriocytes during all developmental stages--*Hamiltonella *and *Wolbachia *in *B. tabaci*, and *Hamiltonella *and *Arsenophonus *in *T. vaporariorum*, while others were located inside and outside the bacteriocyte--*Rickettsia *and *Cardinium *in *B. tabaci*. Symbionts that are strictly localized to the bacteriocytes are vertically transmitted and thus they may contribute to their host's fitness [51]. However, they are less likely to be able to manipulate their host's reproduction since this requires invading reproductive organs outside the bacteriocyte. Thus, the restricted localization of *Hamiltonella *in both *B. tabaci *and *T. vaporariorum*, *Wolbachia *in *B. tabaci *and *Arsenophonus *in *T. vaporariorum *suggests their involvement in providing the host with a functional advantage rather than in manipulating its reproduction. Interestingly, *Wolbachia *was localized to the bacteriocyte and was not observed outside it, invading other organs. *Wolbachia *can be found in all major insect orders at various different frequencies, and it has been associated with reproductive disorders [16]. However, the localization pattern in *B. tabaci *observed here suggests that *Wolbachia *does not manipulate reproduction in this whitefly, but rather performs other unknown functions. It cannot be excluded that at some stages of the adult development, *Wolbachia *may invade the reproductive system and causes known reproductive manipulations, however, discovering this requires more investigations. One major advantage of the confined localization of some symbionts with the primary symbiont in the bacteriocyte is that the host immune system is thus avoided, representing a bidirectional advantage for the host which invests fewer resources in maintaining the symbiont levels and for the symbiont, which is not recognized by the immune system of the host. This confined localization ensures low cell numbers of the bacterium because of the limited space in the bacteriosome, and thus for the host, a lower fitness cost is associated with maintaining the symbiont. An additional advantage for the symbiont is the ease of vertical transmission from one generation to the next. "Hitching a ride" with the primary symbiont in the bacteriocyte exempts the secondary symbiont from invading and entering the egg alone during oogenesis, and ensures its transmission during the transfer of the bacteriocyte to the egg [16].

The localization pattern of the secondary symbionts confined to the bacteriocyte in both *B. tabaci *and *T. vaporariorum *showed some specific localization to patches. This localization pattern was consistent in all of the individuals tested, and suggests specific sharing inside the bacteriocyte, with each symbiont, primary and secondary, occupying its own niche. Interestingly, all of the symbionts detected in *B. tabaci *were found to co-exist in the same individual, in varying percentages, suggesting little or no competition for space, with the exception of *Arsenophonus *and *Hamiltonella *which were not found together in *B. tabaci*, although they were found together in *T. vaporariorum*. Interestingly, in this latter species, their localization pattern in the bacteriocyte looked exactly the same, suggesting localization in exactly the same places or one inside the other [52]. Future experiments using TEM and ultrastructural localization should shed more light on the exact location of these symbionts relative to one another.

In contrast to the symbionts that were restricted to the bacteriocytes, *Rickettsia *and *Cardinium *in *B. tabaci *showed a scattered localization pattern and were seen outside the bacteriocyte. These two symbionts are known to manipulate host reproduction in many arthropods [53,54], and this fits well with their localization pattern in *B. tabaci*. Previously, *Rickettsia *has been shown to exhibit two different localization phenotypes: scattered throughout the body and confined to the bacteriocyte [22]. These two phenotypes were never observed together in the same individuals. It is not clear whether these localization phenotypes are characteristic of the host or if they are due to different bacteria localizing differently in the host's body. Our FISH results showed the presence of both scattered and confined phenotypes in the same individuals for *Rickettsia *(Figure 10), and *Cardinium *(Figure 8). These phenotypes are similar to the obligatory *Rickettsia *in booklice, in which it was found to appear with both phenotypes in the same individual [55]. We further observed concentration of *Rickettsia *at the circumference of the bacteriocyte, suggesting a stage in which *Rickettsia *concentrates around the developing oocytes for entry, for transferral to the next generation.

## Conclusions

Our study describes the distribution of two whitefly species in Croatia and their infection and co-infection status by secondary symbionts. Co-infections revealed a unique pattern of co-sharing the bacteriocyte by the primary and different secondary symbionts. Co-sharing of the same cell by multiple symbionts while maintaining infections over time by vertical transmission through the egg is unique in whiteflies. This sharing provides a unique system to study interactions among bacteria that co-inhabit the same cell. Positive and/or negative interactions among these symbionts--cooperation and antagonism--are part of the multiple interactions that one can expect within their small niche. Competition between symbionts for space and resources may affect their small environment and their host. The host can be affected through competition between the primary and secondary symbionts within the bacteriocyte. Such microbial diversity provides a unique opportunity for artificial interference and manipulation to disrupt this diverse community as a better means of controlling whiteflies, which are major pests in many agricultural systems.

## Methods

### Whitefly collections

Populations of the sweet potato whitefly *B. tabaci *and the greenhouse whitefly *T. vaporariorum *were collected during the years 2008-2009 across Croatia. Attempts were made to include populations from all parts of the country, but in some areas, no whiteflies could be found. In addition, three populations were collected from Bosnia and Herzegovina, and one population from Monte Negro for comparison with nearby countries. The whiteflies were collected from the plants into glass Pasteur pipettes attached to a mechanical hand-held aspirator. Each collected population in each location was collected from different leafs on different plants. Some of the populations were collected in greenhouses, and some in open fields and private gardens. Table 1 shows a list of the collected whitefly populations from the different locations and the host plants on which these populations were collected. After collection, all adult individuals were immediately transferred to absolute ethanol for preservation and were kept at room temperature until processing for secondary-symbiont screening.

### Whitefly population rearing

After collection from the field, three whitefly populations (Zadar, Kastela, Turanj) were directly transferred as adults to insect-proof cages containing cotton cv. Acala seedlings (obtained from Zeraim Gedera, Israel). These adults were given a week to lay eggs and to establish a colony. The colonies were then maintained in the laboratory under standard conditions (26 ± 2°C, 60% RH, 14/10 h of light/dark).

### Identification of *B. tabaci *biotypes

Biotypes were identified using microsatellite markers with the primer pair Bem23 which distinguishes between B and Q biotypes based on the fragment size amplified [56]. Another method was used to verify the B and Q biotypes which consisted of sequencing a fragment of the mitochondrial (mt) COI gene after amplification by PCR. The PCR conditions for amplifying mtCOI and the microsatellite markers were as previously described [11], and the primer sequences are given in Table 2.

### Screening for the presence of secondary symbionts

Whiteflies (*n *= 10-20) were individually analyzed for the presence of secondary symbionts and for biotype determination. Genomic DNA from each whitefly was isolated in lysis buffer as previously described [11,57]. The same DNA from each individual was used to screen for the presence of all potential symbionts and for biotype. The presence of *Hamiltonella*, *Rickettsia*, *Wolbachia*, *Arsenophonus*, *Cardinium *and *Fritschea *in the samples was determined using genus-specific primers for amplifying 16S or 23S rDNA gene fragments (Table 2). PCRs were carried out as previously described [11]. PCR products were visualized on 1.5% agarose gel containing ethidium bromide. To verify the identity of the PCR products, bands were excised from the gel and DNA was isolated from them and sent for sequencing (ABI 3700 DNA analyzer, Hylabs, Rehovot, Israel). The resulting sequences were run against the non-redundant nucleotide database using the BLAST algorithm of the National Center for Biotechnology Information (NCBI).

### Fluorescent in situ hybridization analysis

FISH analysis of adults, nymphs and eggs was performed as previously described [22] using short symbiont-specific 16S/23S rRNA DNA probes harboring a fluorescent Cy3/Cy5 molecule on their 5' end (Table 3). Absence of cross hybridizations and probe specificity was tested using the "probe match" analysis tool in the Ribosomal Database Project II http://rdp.cme.msu.edu/. Stained samples were mounted whole and viewed under an IX81 Olympus FluoView 500 confocal microscope (Olympus, Tokyo, Japan). For each developmental stage, at least 50 specimens were viewed under the microscope to confirm reproducibility. Optical sections(0.7-1.0 μm thick) were prepared from each specimen. Specificity of detection was confirmed using no probe staining and RNase-digested specimen staining. In addition, each population was tested with all of the probes listed in Table 2 as controls. Thus, staining of a population known not to have a particular symbiont but harboring others was performed.

**Table 3:** List of probes used for FISH in this study

Target symbiont	Probe name and dye	Sequence (5'-> 3')	Reference
*Portiera*	BTP1-Cy3	TGTCAGTGTCAGCCCAGAAG	[9]
*Rickettsia*	Rb1-Cy5	TCCACGTCGCCGTCTTGC	[9]
*Hamiltonella*	BTH-Cy5/Cy3	CCAGATTCCCAGACTTTACTCA	[22]
*Cardinium*	Card-Cy5	TATCAATTGCAGTTCTAGCG	[58]
*Arsenophonus*	Ars2-Cy5	TCATGACCACAACCTCCAAA	[22]
*Wolbachia*	W1-Cy5	CTTCTGTGAGTACCGTCATTATC	[33]

## Authors' contributions

MS performed the experiments. SK participated in rearing the whitefly populations and performing some of the experiments. MS, KZ, SGB and MG collected whitefly populations in Croatia. MG and MS designed the study. MG drafted the manuscript. All authors have read and approved the final manuscript.
